# Localized delivery of brain-derived neurotrophic factor from PLGA microspheres promotes peripheral nerve regeneration in rats

**DOI:** 10.1186/s13018-022-02985-x

**Published:** 2022-03-18

**Authors:** Zheng-liang Shi, Zhi-yong Fan, Hua Zhang, Shen-tai Li, He Yuan, Jiu-hui Tong

**Affiliations:** Department of Orthopedics, The Second Hospital of Hebei Medical University, No. 215, Hepingxi Road, Shijiazhuang, 050000 Hebei Province China

**Keywords:** PLGA microsphere, BDNF, Tissue engineering, Peripheral nerve regeneration

## Abstract

**Background:**

Repair of peripheral nerve defect presents a considerable challenge for reconstructive surgeons. The aim of this study is to develop a brain-derived neurotrophic factor (BDNF) from poly(D,L-lactide-co-glycolide) (PLGA) microspheres for the treatment of the peripheral nerve defect.

**Method:**

BDNF microspheres were prepared by using an oil-in-water emulsification-solvent evaporation method. The morphology, particle size, encapsulation efficiency, drug loading and sustained release performance of microspheres was observed and calculated. Adipose mesenchymal stem cells (ADSCs) were isolated and expanded. ADSCs were divided into four groups: control, BDNF, blank microsphere and BDNF microsphere groups. Cell count kit-8 (CCK-8) assays were used to assess cell proliferation. Cell migration was determined by Transwell assays. Twenty-eight male Sprague–Dawley rats underwent transection damage model on the right sciatic nerve. The wet weight ratio of the gastrocnemius muscle was calculated by comparing the weight of the gastrocnemius muscle from the operated side to that of the normal side. Neuroelectrophysiological testing was performed to assess nerve function recovery. Nerve regeneration was evaluated by histological analysis and immunohistochemical staining.

**Results:**

The microspheres were spherical and had uniform size (46.38 ± 1.00 μm), high encapsulation efficiency and high loading capacity. In vitro release studies showed that BDNF-loaded microspheres had good sustained release characteristics. The duration of BDNF release was extended to more than 50 days. BDNF or BDNF microsphere promote the proliferation and migration of ADSCs than control group (*P* < 0.05). Compared with control group, BDNF significantly decreased the nerve conduction velocity (NCV) and compound amplitude (AMP) (*P* < 0.05). The nerve fibers in the BDNF microsphere group were closely arranged and uniformly distributed than control group.

**Conclusion:**

BDNF/PLGA sustained-release microsphere could promote the migration of ADSCs and promoted neural differentiation of ADSCs. Moreover, BDNF/PLGA sustained-release microsphere ameliorated nerve conduction velocity and prevented neuralgic amyotrophy.

## Background

Peripheral nerve injury is a common clinical problem, and severe nerve injury has a devastating impact on the quality of life of patients. In recent years, the incidence of chronic wounds with defect to peripheral nerves has increased [1, 2]. Repair of peripheral nerve defect presents a considerable challenge for reconstructive surgeons [3–5]. Although peripheral nerves exhibit greater regenerative abilities after nerve injury as compared with central nerves, peripheral nerve injury, especially peripheral nerve injury with long nerve defects, is still a severe health problem.

At present, end-to-end anastomosis surgery is often used for shorter nerve defects, and the clinical gold standard for longer nerve defects is autologous nerve transplantation. However, the limitations of autografts, including insufficient sources, morbidity at donor site and necessity for multiple surgeries, have greatly hampered their clinical application. Given the slow rates of nerve regeneration, tissue adhesion, and muscle atrophy, obtaining satisfactory results for impaired nerve function is difficult.

Brain-derived neurotrophic factor (BDNF) is one of the most widely distributed neurotrophic factors in the brain, which supports growth and survival of neurons [6, 7]. BDNF is a member of the trophic factors of nerve regeneration microenvironment and therefore involved in the regulation of nerve fiber regeneration and protection of neurons [8].

BDNF promotes the survival and differentiation of a variety of neurons in the central and peripheral nervous systems [9].

BDNF has achieved good clinical results, but the in vivo stability is poor; they are vulnerable to degeneration or inactivation and are easily diminished in the blood circulation [10].

It was hypothesized that constructing a controlled-release system may prolong the action time and improve the bioavailability of exogenous BDNF.

Biodegradable nanospheres generated from a biocompatible polymer, poly(D,L-lactide-co-glycolide) (PLGA), have been studied extensively as implantable reservoirs for sustained-release drug delivery [11, 12]. Administration PLGA microsphere not only improves patient compliance, but also the therapeutic efficacy of BDNF [13]. Therefore, if BDNF was successfully incorporated into the PLGA microsphere, constructed BDNF-PLGA microspheres could potentially be uses clinical practice.

In this study, we fabricated BDNF-PLGA microspheres and identify that localized delivery of BDNF from PLGA microspheres promotes peripheral nerve regeneration in rats. We hypothesized that BDNF-PLGA microspheres could greatly promote peripheral nerve regeneration and have a potential application in nerve regeneration.

## Materials and methods

### Preparation of the BDNF-PLGA microspheres

BDNF was dissolved in deionized water (µL) for the internal water phase, and PLGA was dissolved in dichloromethane to prepare a PLGA solution (60 g/L) as the oil phase. Polyvinyl alcohol was dissolved in deionized water to prepare an aqueous solution (10 g/L) as the external water phase. Add internal water phase (100 μL) to 2 mL of oil phase to shake for 30 s to form a primary emulsion use in ice bath. Then, the primary emulsion was added to 20-mL outer water phase and 500 r/min magnetic stirring for 24 h to remove the dichloromethane to obtain a microsphere solution. Then, the microsphere solution was centrifuged at 2000 r/min for 3 min in a 16-cm centrifuge. The supernatant was discarded to get the initial micro-ball which was washed 3 times with deionized water and then freeze-dried at -20 °C for 24 h.

### Morphology observation and particle size distribution of BDNF/PLGA microspheres

Appropriate amount of BDNF/PLGA microspheres fixed on the conductive glue, the morphological characteristics of the microspheres were observed using a scanning electron microscope after spraying gold treatment. The software nanomeasure1.2 is used to analyze the particle size distribution of the microspheres (*n* = 127).

### Encapsulation efficiency and drug loading of BDNF/PLGA microspheres

The supernatant after centrifugation of the microsphere solution was obtained, and the absorbance of the supernatant was measured by ELISA.

In brief, 50 μl of standard or serum with sample diluent was added to a microtiter plate that had been pre-coated with the corresponding antibody and incubated at 37 °C for 30 min. The antibody-enzyme conjugate was then added, and the plate was incubated for 30 min at 37 °C and washed. After washing, 50 μl of chromogenic reagent A and 50 μl of chromogenic reagent B were added to the microtiter plate. The plate was incubated at 37 °C for 10 min and protected from light. Finally, 50 μl of stop solution was added to terminate the reaction. The absorbance was measured at 450 nm using a microplate reader.

According to the standard curve equation, the BDNF concentration in the supernatant was calculated.

The encapsulation amount and encapsulation efficiency of the BDNF sustained-release microspheres are calculated using the following formula: Encapsulation rate = mass of BDNF in microspheres/total mass BDNF × 100%. Drug loading (µg/g) = BDNF mass in microspheres /microsphere mass. The mass of BDNF in the microspheres = Total mass of BDNF—BDNF mass in the supernatant.

### In vitro release of BDNF/PLGA microspheres

A total of 5 mg of the BDNF/PLGA microspheres (the drug loading is 37.44 µg/g) powder was placed in a 1.5-mL EP tube. Then, 1-mL PBS solution (0.1%) was placed in a constant temperature shaker at 37 °C at 60 r/min shake. The supernatant was collected at 0, 1, 4, 7, 10, 20, 30, 40 and 50 d. The content of BDNF in the supernatant was determined by ELISA.

### Isolation and culture of adipose-derived mesenchymal stem cells (ADSCs)

Infrapatellar fat pad in the knee joint cavity of New Zealand white rabbits (1 month) was extracted according as established previously [14]. In brief, fat were cut into small pieces (~ 2 mm) and enzymatically dissociated using a 0.05% collagenase II solution (Worthington, Columbus, OH, USA) for 30 min at 37 °C. After neutralization of the enzyme, cells were centrifuged at 500×*g* for 5 min and filtered through a 70-μm nylon mesh (Merck Millipore, Danvers, MA). ADSCs at passage 3 were used. ADSCs were characterized using flow cytometry with stem cells markers: CD105, CD45, CD73 and CD34. Moreover, ADSCs were characterized based on their adipogenic, osteogenic, and chondrogenic capacities.

### The effect of BDNF/PLGA microspheres on cell proliferation and migration

The third-generation ADSCs were seeded in 96 wells at a density of 3000/well. After removing the medium, add different groups of medium. The experiment is divided into six groups, and DMEM/F12 medium (negative control), BDNF medium, PLGA microsphere, and PLGA microsphere, containing 10, 100, 1000 g/L BDNF. The drug loading of PLGA microspheres was 194.80 µg/g. After culturing in an incubator with a volume fraction of 5% CO_2_ at 37 °C for 1, 3 and 5 days. Then, 10 μL of CCK-8 solution was added to the wells and then incubated for another 2 h and measure in a 450-nm microplate reader. Each sample was repeated 5 times, and the results were averaged.

Cell migration was performed as follows: 0.1 mL (5 × 10^4^ cells) of ADSCs are added to the upper chamber and incubated in a 37 °C incubator 24 h. After washing with water, the non-migrated cells were scraped from the upper chamber with cotton swab, and migrated cells were fixed with 4% paraformaldehyde, and crystal violet (0.1%) was used to stain cells for 10 min. Bottom surface was then counted with an inverted microscope. The titration was repeated three times for each sample, and the results were averaged. The researcher who performed the cell counts was blinded to the treatment groups.

### Animal experiment

The rats were specific-pathogen-free (SPF) grade animals, and were fed in a barrier SPF environment under 12-h light/dark cycle. All rats were fasted for 24 h before surgery, and this study protocol was conducted in accordance with the Guidance Suggestions for the Care and Use of Laboratory Animals, formulated by the Ministry of Science and Technology of China. A total of 28 male Sprague–Dawley rats weighing 300 ± 50 g were randomly divided into four groups (Saline (*n* = 7), PLGA (*n* = 7), BDNF (*n* = 7) and PLGA/BDNF (*n* = 7)). In brief, rats were anesthetized with an injection of a mixture of chloral hydrate (0.4 g/kg ip). Rats were placed in the prone position on the operating table following back shaving and routine disinfection.

An oblique incision approximately 3-cm long was made along the posterior lateral side of the left thigh to expose the left sciatic nerve. The sciatic nerve defect model was prepared by cutting and excising the sciatic nerve approximately 1-cm long at the lower margin of the piriformis muscle 5 cm away as previously described [15]. The nerve defect was repaired with different methods: group A (autogenous nerve transplantation), the nerve defect was repaired with autogenous nerve that suture in situ; group B (autogenous nerve transplantation plus PLGA microsphere), the nerve defect was repaired with an acellular nerve graft and Schwann cell-like cells that differentiated from ADSCs; group C (autogenous nerve transplantation plus BDNF) and group D (autogenous nerve transplantation plus PLGA/BDNF). Following confirmation of the successful completion of anastomosis, 100-µl BDNF-PLGA microspheres (20 µg), 100-µl BDNF (20 µg) or 100-µl PBS was injected into the silicone tube using a microinjector to bridge the nerve gap. After full recovery from anesthesia, the animals were returned to the animal house.

### Sciatic functional index (SFI) evaluation

SFI was measured at 28 days after surgery as described previously [16]. Rats were restrained by fixing the forelimbs to a wooden board, and the hind limbs only fixed the operation sides. The length of the normal side print length (PL), toe spread (TS) and intermediary toe spread (IT) was measured using a ruler. The formula of SFI: SFI = − 38.8 × [(EPL − NPL) /NPL] + 109.5 × [(ETS − NTS)/NTS] + 13.3 × [(EIT − NIT)/NIT] − 8.8. EPL = PL of operated side; NPL = PL of nonoperated side; ETS = TS of operated side; NTS = TS of nonoperated side; EIT = IT of operated side; NIT = IT of nonoperated side. An SFI of 0 means that the nerve function is normal, -100 means that the nerve function is complete all lost.

### Neuroelectrophysiological testing

Sciatic nerves were isolated from rats at the week 12 of the experiment. The stimulating electrode was placed sequentially in the proximal and distal sciatic nerves and electrode spaced 10 mm apart. The recording electrode was placed in the triceps surae. Nerve conduction velocity (NCV) and compound amplitude (AMP) were recorded through Medlec Synergy Electrophysiological System (Oxford Instrument Inc., Oxford, UK).

### Wet weight ratio of gastrocnemius

The bilateral gastrocnemius muscles were completely separated, and the wet weight ratio of the gastrocnemius muscle was weighed with an electronic balance. The wet weight ratio of the gastrocnemius muscle was calculated by comparing the weight of the gastrocnemius muscle from the operated side to that of the normal side.

### Histological evaluation

A total of three investigator performed histological evaluation, and they were blinded to treatment groups. Tissue samples were post-fixed in zinc solution, dehydrated, embedded in paraffin, sectioned and processed for histological examination. H&E and Toluidine blue staining were then performed to count the number of myelinated nerve fibers. Immunofluorescence (IF) of S-100 and GFAP were carried out as we described previously [17]. A total of five sections were analyzed for each sample and the mean value computed for each specimen.

### RT-qPCR

Total RNAs from BMSCs were separated by TRIzol reagent (Invitrogen) and reverse transcribed into complementary DNA by BestarTM qPCR RT kit (DBI Bioscience, China). RTqPCR was conducted using BestarTM qPCR MasterMix kit (DBI Bioscience) on an ABI Prism 7500 Real-Time PCR System (Applied Biosystems, USA) following the manufacturer’s instructions. GFAP and S100 expression was normalized to GAPDH. The relative expression of RNAs was calculated by the 2^−ΔΔCt^ method. Primers were designed by Prime 5.0.

### Statistical analysis

SPSS 21.0 statistical software (SPSS Inc., Chicago, IL, USA) was used for statistical analysis. In this study, continuous data were used as the mean ± standard deviation (SD), and the one-way ANOVA with Bonferroni's test post hoc analysis for multiple comparisons was performed for statistical analysis and used for comparison. Seven independent samples were used for each experiment. *P* < 0.05 was considered statistically significant.

## Results

### Morphology and size distribution

**Fig. 1:**
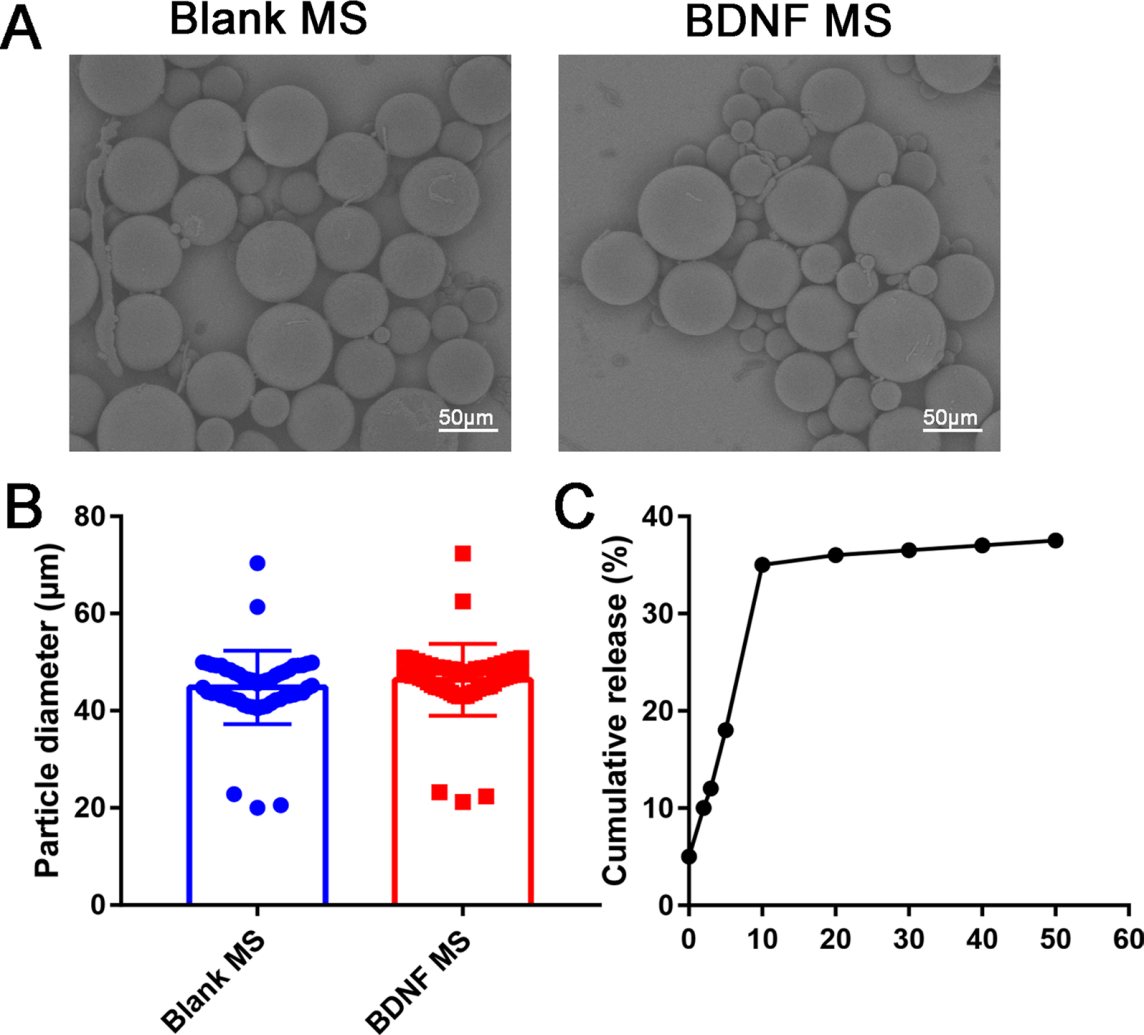
**A** Scanning electron microscope (SEM) images of blank PLGA microspheres and BDNF-loaded PLGA microspheres; **B** particle diameter of blank PLGA microspheres and BDNF-loaded PLGA microspheres; **C** cumulative in vitro release profile of BDNF from PLGA microspheres

Scanning electron microscopy showed that the microspheres had uniform size and smooth surface. Microspheres had overall smooth and nonporous surfaces (Fig. 1A). The diameter of the blank microsphere was 44.81 ± 1.02 μm, and the diameter of the BDNF microsphere was 46.38 ± 1.00 μm. There was no significant difference between the blank microsphere and BDNF microsphere (Fig. 1B**, ***P* > 0.05). As shown in Fig. 1C, the cumulative release of BDNF/PLGA microspheres reached 30 ng within the first week, then the BDNF/PLGA microspheres are slowly released into a slow linear release period, which can be released slowly up to 50 days.

### Encapsulated ratio and drug loading efficiency

Compared with 0.5 μg BDNF, when BDNF increased, the encapsulated ratio of BDNF was increased. When BDNF increased to 20 μg, the encapsulated ratio of BDNF did not overtly alter. Overall, PLGA microspheres are all show high encapsulation efficiency and drug loading, and their encapsulation efficiency is above 90% (Table 1).

**Table 1:** Encapsulation efficacy and encapsulation dose of copolymer microspheres between different concentrations of BDND (*n* = 3)

BDNF (μg)	Encapsulated ratio (%)	Drug loading efficiency (μg/g)
0.5	94.85 ± 0.35	9.52
1	97.66 ± 0.28	19.28
2	93.85 ± 0.36	35.48
5	97.86 ± 0.52	97.56
10	97.28 ± 0.34	195.26
20	96.59 ± 0.26	380.34

### Cell viability and migration ability

**Fig. 2:**
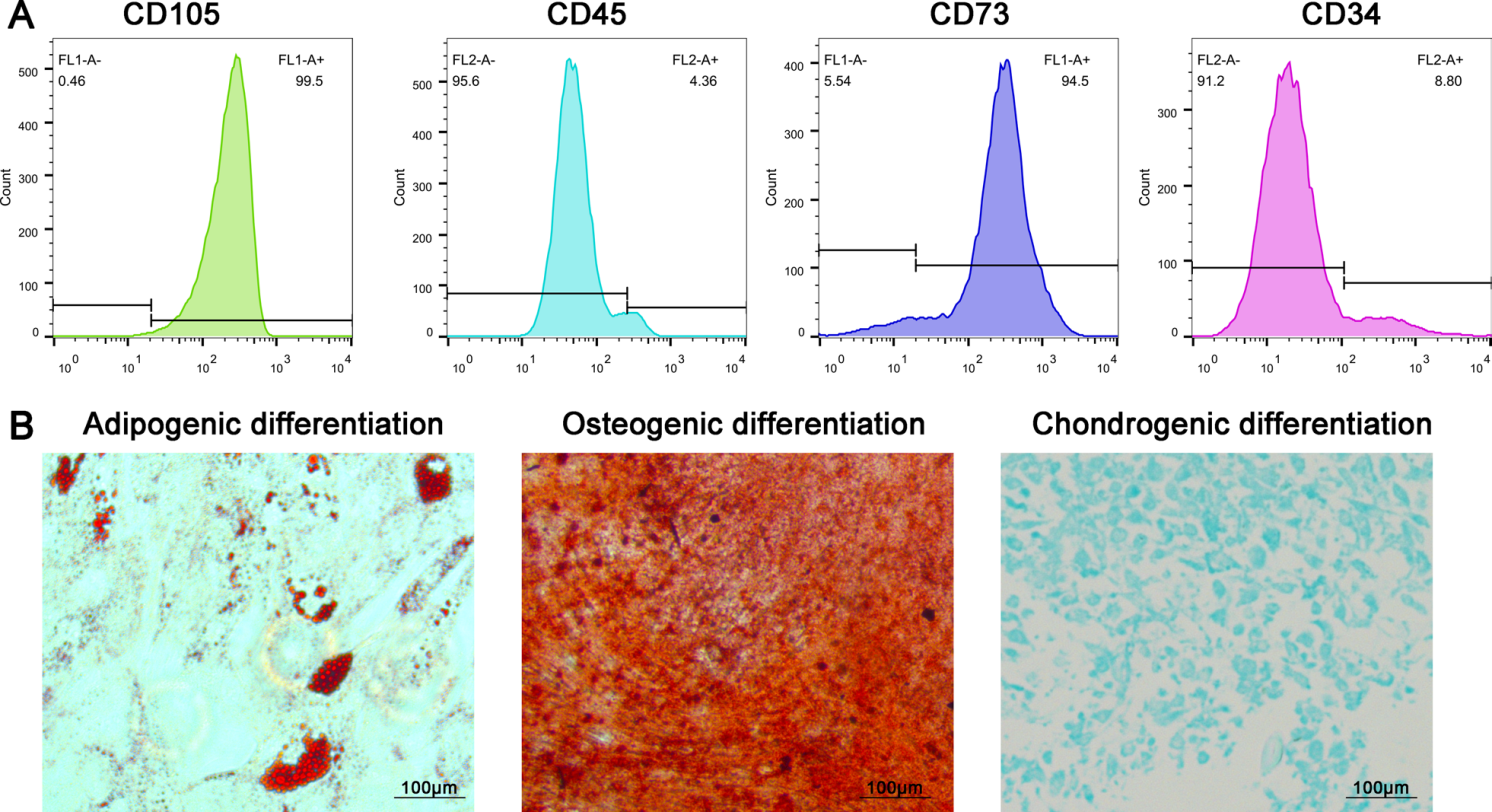
**A** Phenotypic characterization by flow cytometry of the following markers: CD105, CD45, CD73 and CD34; **B** trilineage differentiation capability of ADSCs cultured in adipogenic, osteogenic and chondrogenic medium, respectively

The flow cytometer illustrated that over 95% of ADSCs expressed CD90 and CD73; however, few ADSCs expressed CD34 and CD45 (Fig. 2A). ADSCs at passage 3 exhibited adipogenic, osteogenic and chondrogenic differentiation capacity (Fig. 2B).

**Fig. 3:**
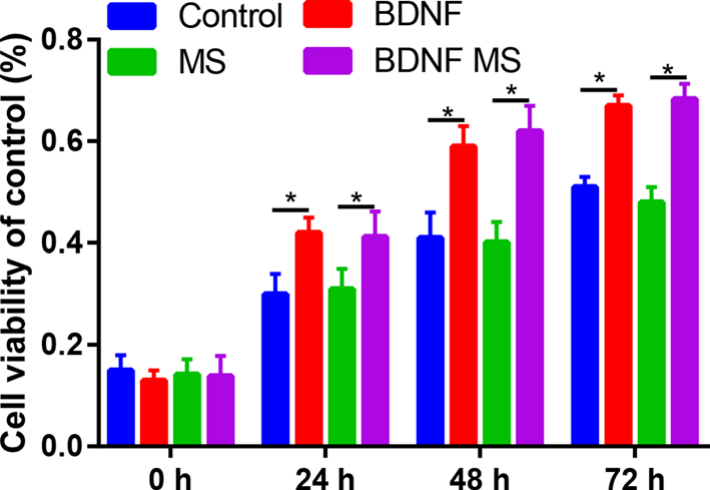
Cell viability of the ADSCs in control, BDNF, MS and BDNF MS groups. *P < 0.05

**Fig. 4:**
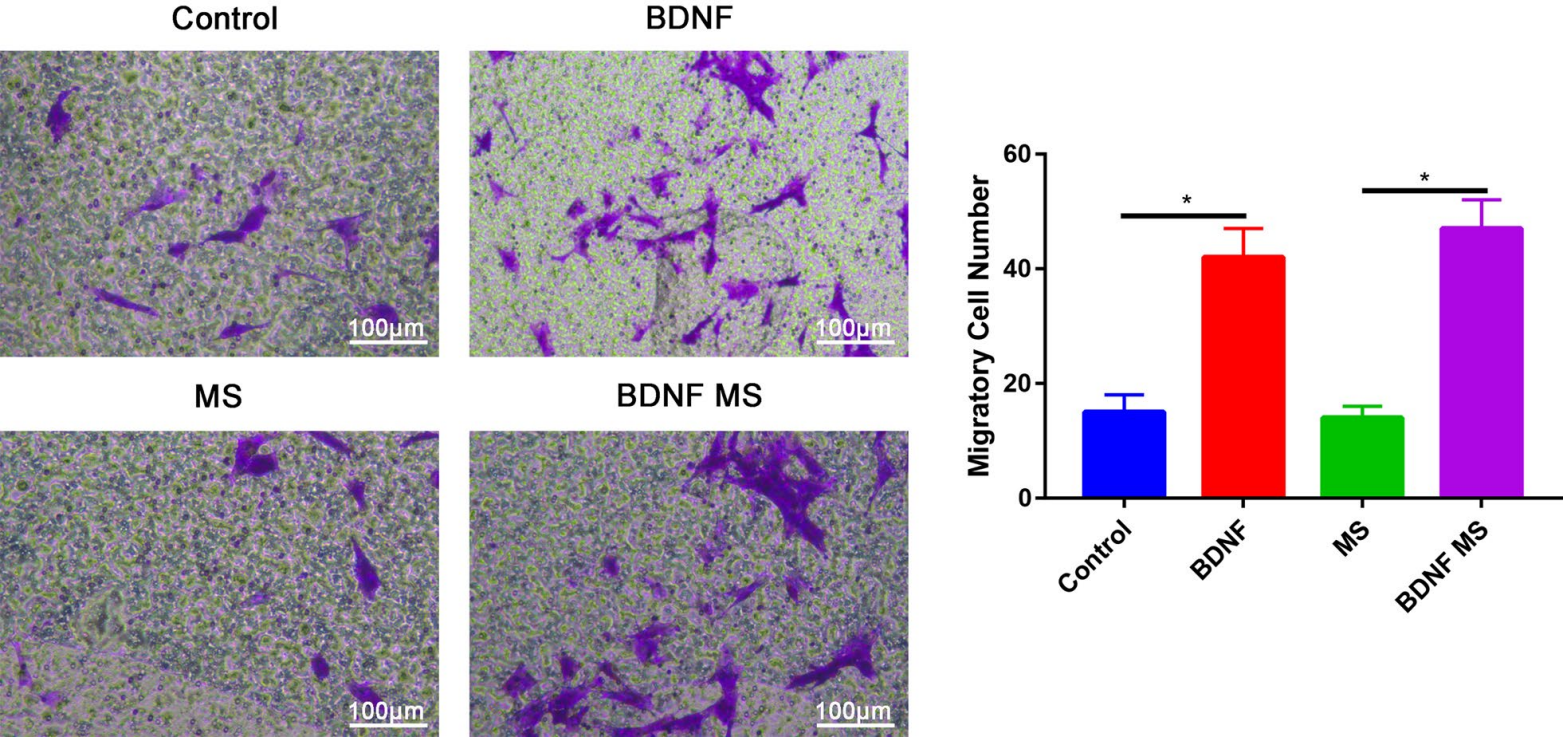
Cell migratory of the ADSCs in control, BDNF, MS and BDNF MS groups. *P < 0.05

After 1, 3 and 5 days of culture, the number of cells increased significantly. Compared with the control group, the proliferation of ADSCs significantly increased following BDNF or BDNF microspheres at 24, 48 and 72 h (Fig. 3). Compared with control or microspheres groups, BDNF or BDNF microspheres groups significantly increased the migratory cells (Fig. 4).

### Real-time PCR

**Fig. 5:**
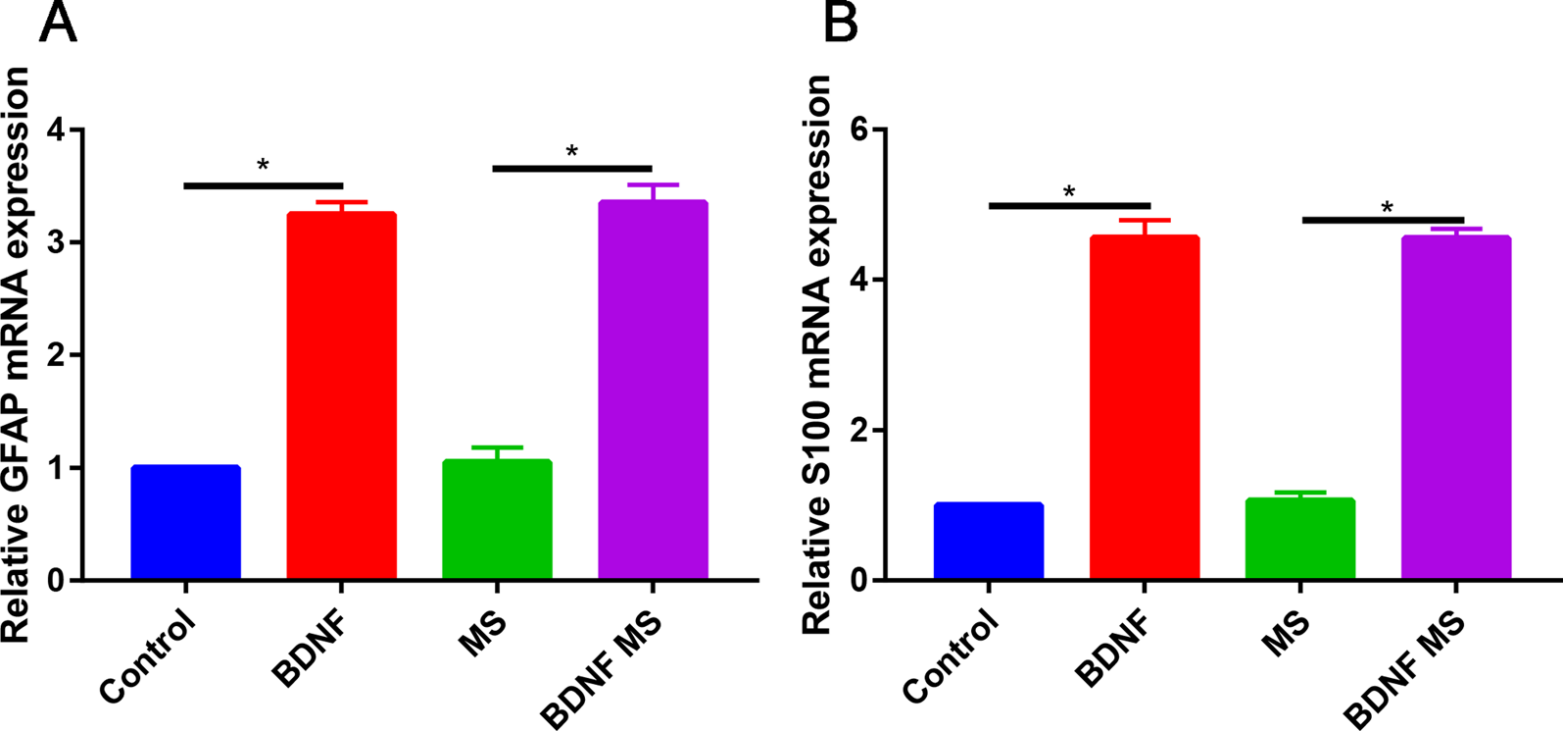
**A** Relative mRNA expression of GFAP in control, BDNF, MS and BDNF MS groups; **B** relative mRNA expression of S100 in control, BDNF, MS and BDNF MS groups. *P < 0.05

Compared with control group, BDNF or BDNF microspheres significantly increased the GFAP (Fig. 5A) and S100 expression (Fig. 5B).

### Neuroelectrophysiological results

Compared with control group, BDNF significantly decreased the NCV, and the difference was statistically significant (Table **2**, *P* < 0.05). Compared with microspheres, BDNF microspheres significantly decreased the NCV (Table 2**,** *P* < 0.05). Compared with control group, BDNF significantly decreased the AMP (Table 2**,** *P* < 0.05). Compared with microspheres, BDNF microspheres significantly decreased the AMP (Table 2**,** *P* < 0.05). The SFIs of rats at 8 weeks after surgery are shown in Table 3.

**Table 2:** Neuroelectrophysiological results of control, BDNF, MS and BDNF MS

Index	Control	BDNF	MS	BDNF MS
NCV (m/s)	36.25 ± 4.34	28.85 ± 3.56	36.89 ± 2.56	26.58 ± 2.11
AMP (mv)	5.52 ± 1.23	3.76 ± 1.14	5.34 ± 1.87	3.56 ± 1.06

**Table 3:** SFI of the different treatment groups

Index	Control	BDNF	MS	BDNF MS
SFI	− 52.31 ± 2.26	− 22.69 ± 3.14	− 35.25 ± 1.29	− 23.85 ± 2.55

Longitudinal comparisons of each group showed that the SFI indexes of groups BDNF microspheres and BDNF were significantly better than those of control and microsphere group at 8 weeks after surgery (*P* < 0.05).

**Fig. 6:**
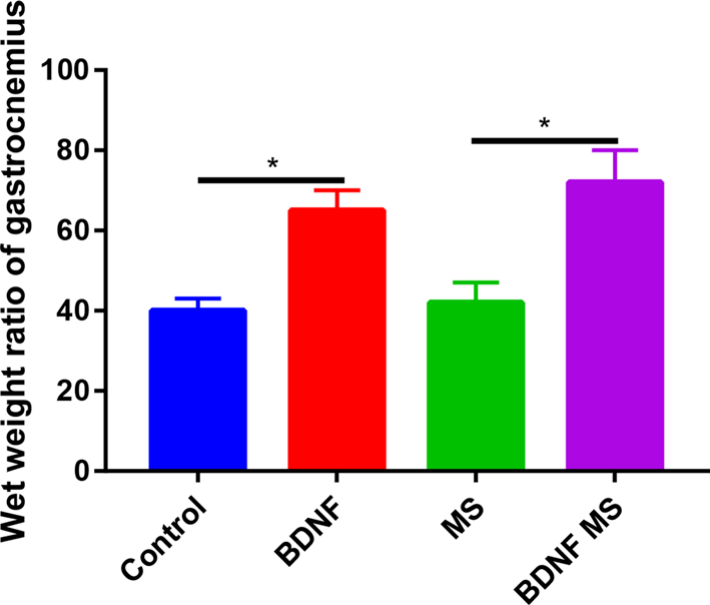
Wet weight ratio of gastrocnemius in control, BDNF, MS and BDNF MS groups. *P < 0.05

BDNF or BDNF microsphere significantly increased the wet weight ratio of gastrocnemius than control and microspheres groups respectively (Fig. 6).

### Histological evaluation

**Fig. 7:**
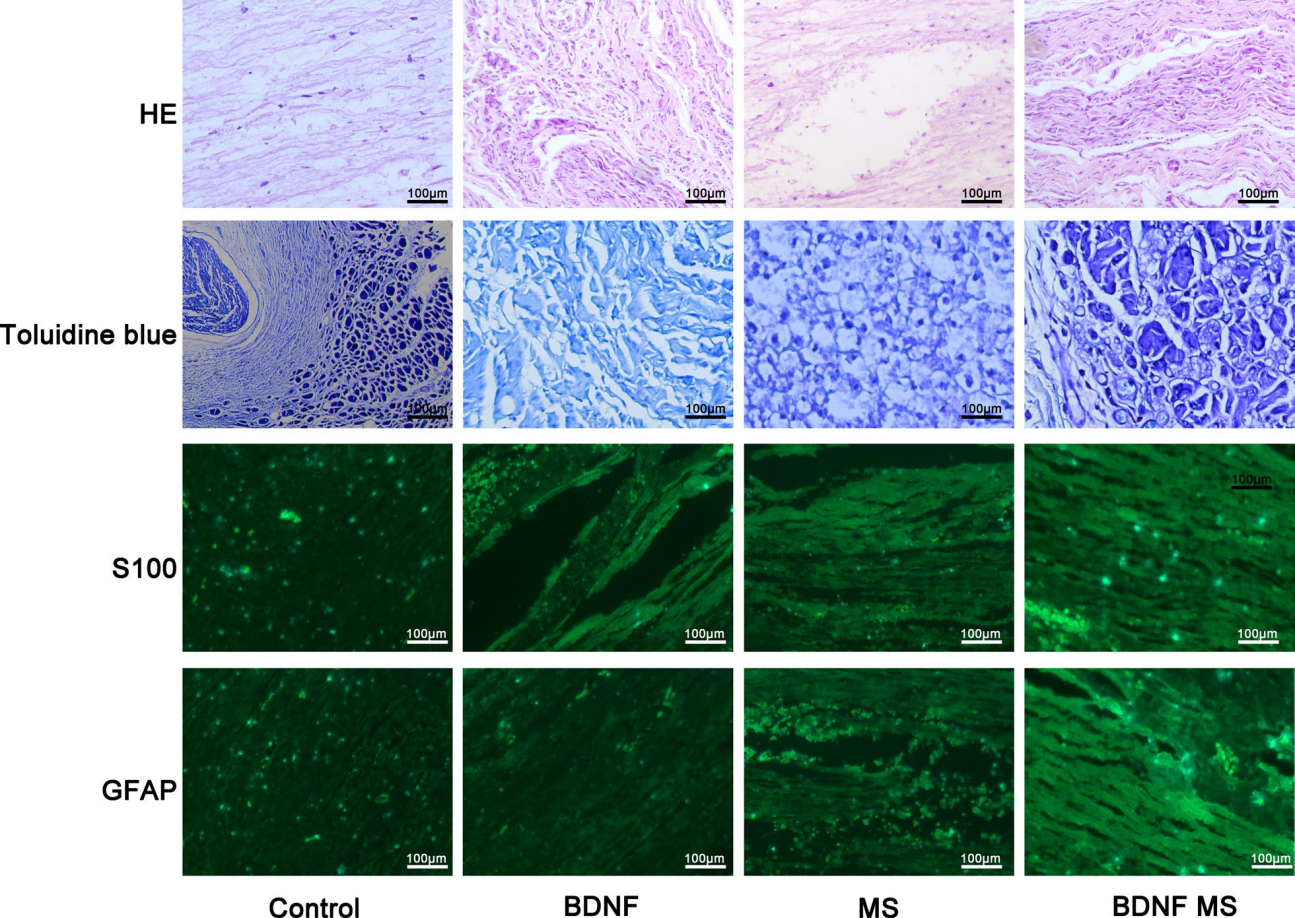
Histological observation at 8 weeks after surgery. HE staining, Toluidine blue staining, S100 and GFAP immunofluorescence staining of longitudinal sections of control, BDNF, MS and BDNF MS groups, respectively

Eight-week post-surgery, HE staining revealed that the inner basal layer and the nerve fiber structure were intact in all groups. The newly formed nerves appeared in a wave-like pattern, and the cells were distributed within the nerve fibers. Compared with control group, BDNF and BDNF MS groups produced more newly formed nerves. For toluidine blue stained, the myelin of regenerated myelinated nerve fibers in control group was dense, and morphology and diameter were irregular. Following treatment with BDNF or BDNF microsphere, the numbers of myelinated nerve fibers were increased and the morphology was regular. Immunofluorescence of S100 and GFAP were positively expressed in each group, which indicated that the formed tissue was nerve tissue. And the level of S100 and GFAP expression in the BDNF or BDNF microsphere groups were significantly higher than that in the control or blank microsphere groups (Fig. 7).

## Discussion

The BDNF-PLGA microspheres employed in the present study were able to gradually release BDNF in the early phase of sciatic nerve regeneration in rats, which leads to accelerated nerve regeneration and functional recovery. The use of BDNF-PLGA microspheres may be a promising and effective approach for peripheral nerve regeneration.

PLGA is a biodegradable polymer approved by the FDA that undergoes hydrolytic degradation in living organisms with harmless degradation products [18–20]. BDNF enhances neurogenesis and neurotransmission across the synapses, promotes synaptic growth and modulates synaptic plasticity [21]. BDNF is rapidly degraded by proteases in the body following direct administration and cannot maintain effective concentration and sustained release time [22]. We successfully developed BDNF-loaded PLGA microspheres which overcome these disadvantages of BDNF direct administration and elevates its biological effects.

PLGA is widely used in drug research due to its biocompatibility, biodegradability and multidimensional degradation kinetics [23, 24]. Park et al. [25] constructed PLGA microsphere matrix containing TGF-β3 for neocartilage formation. They concluded that growth factor delivery of PLGA microsphere can be used to engineer synthetic extracellular matrix. What’s more, single intra-articular injection of fluvastatin-PLGA microspheres reduces cartilage degradation in rabbits with experimental osteoarthritis [26]. PLGA microspheres as a carrier solves the problem of short half-life of BDNF and easy degradation in vivo [27].

The results showed that BDNF PLGA microspheres have good spherical morphology, smooth surface and uniform particle size distribution. Moreover, there is no adhesion between the microspheres, making it an ideal drug carrier. This result was consistent with the results of other studies [28, 29].

The encapsulation efficiency of PLGA microspheres measured by the indirect method is all above 90%, showing its advantages as a drug carrier. Yang et al. [30] fabricated the hydrochloride PLGA microsphere and found the encapsulation efficiency ranged from 55.64 to 94.33%. Fu et al. [31] identified that the molecular weight of PLGA affect the encapsulation efficiency of PLGA microspheres. In the initial stage, the transfer of the PLGA microspheres adhered to the surface chemical BDNF first diffuses into the surrounding medium, showing a rapid burst phase, release reached 10 µg/L in the first 3 days. Rapid burst phase plays a role in the promotion of stem cells proliferation and migration. BDNF promoted multiple cells proliferation including trophoblastic cell [32], oligodendrocyte precursors [33] and schwann cells [34]. Zhang et al. [35] also found that BDNF could promoting osteoblast migration through ERK1/2 and Akt signaling pathway. Borghesani et al. [36] revealed that BDNF is directly motogenic for granule cells and provides a directional cue promoting migration from the granule cell layer to the internal granule cell layer.

The in vivo study revealed that BDNF microspheres could improve nerve conduction. BDNF is expressed after peripheral nerve injury to stimulate axonal regeneration. In rats, BDNF supplementation in acute nerve injury is also accompanied by increased axonal sprouting and branch length [37]. Electrophysiological examinations of nerve conduction in rat nerves found that BDNF microsphere significantly increased the NCV and AMP compared with control group. S-100 protein is a marker of newborn Schwann cells [22, 38]. GFAP protein is specifically expressed in unmyelinated Schwann cells of the peripheral nervous system [39, 40]. Schwann cells has strong regenerative potential and can guide the regeneration of axons and myelin [41]. Finally, we assessed the expressions of S100 and GFAP in each group. Results found that the expression in the BDNF or BDNF microsphere groups were significantly higher than that in the control or blank microsphere groups. These results suggested that BDNF microsphere could enhanced the nerve factor secretion and finally promoted nerve conduction function recovery.

BDNF is known to play a critical role in neuroprotection and neuronal plasticity in both central and peripheral nervous system [42]. Previously, Zhang et al. [43] conducted an in vitro study that repairing of peripheral nerve defects with chemically extracted acellular nerve allografts loaded with BDNF-transfected bone marrow mesenchymal stem cells. Results found that combination of BDNF-transfected bone marrow mesenchymal stem cells can greatly improve nerve injury.

The present study had some limitations, as described below. First, the duration of PLGA microsphere degradation in vivo was still unclear. Second, how BDNF modulates downstream signaling pathways is not clearly established. Third, long-term effects of BDNF microsphere for nerve damage repair are unknown. This is an animal study that needs to be translated into large animal and then humans to apply the results of the clinically.

## Conclusion

In summary, the BDNF/PLGA sustained-release microspheres are spherical, the drug is evenly distributed, and has a good slowing release characteristic. BDNF/PLGA sustained-release microsphere could promote the migration of ADSCs and promoted neural differentiation of ADSCs. Moreover, BDNF/PLGA sustained-release microsphere ameliorated nerve conduction velocity and prevented neuralgic amyotrophy. Therefore, BDNF/PLGA sustained-release microsphere has great potential in the field of nerve damage repair.

## Data Availability

We state that the data will not be shared since all the raw data are present in the figures included in the article.

## Abbreviations

BDNF: Brain-derived neurotrophic factor
PLGA: Poly(D,L-lactide-co-glycolide)
ADSCs: Adipose-derived mesenchymal stem cells
CCK-8: Cell count kit-8
SFI: Sciatic functional index
PL: Print length
TS: Toe spread
IT: Intermediary toe spread
ETS: TS of operated side
NTS: TS of nonoperated side
EIT: IT of operated side
NIT: IT of nonoperated side
LAT: Latency
NCV: Nerve conduction velocity
SD: Standard deviation
